# Organic carbon sequestration in sediments of subtropical Florida lakes

**DOI:** 10.1371/journal.pone.0226273

**Published:** 2019-12-13

**Authors:** Matthew N. Waters, William F. Kenney, Mark Brenner, Benjamin C. Webster

**Affiliations:** 1 Department of Crop, Soil and Environmental Sciences, Auburn University, Auburn, Alabama, United States of America; 2 Land Use and Environmental Change Institute, University of Florida, Gainesville, Florida, United States of America; 3 Department of Geological Sciences, University of Florida, Gainesville, Florida, United States of America; Tennessee State University, UNITED STATES

## Abstract

Recent studies have shown that sediments of temperate and tropical lakes are sinks for organic carbon (OC), but little is known about OC burial in subtropical lakes. There are questions regarding the ability of subtropical lakes to store OC, given their relatively warmwater temperatures, lack of ice cover, frequent water-column mixing, and labile carbon forms. We used ^210^Pb-dated sediment cores from 11 shallow Florida (USA) lakes to estimate OC burial, i.e. net OC storage, over the last ~100 years. Shallow Florida water bodies average ~30% OC content in their sediments and displayed rates of net OC accumulation (63–177 g C m^-2^ a^-1^) that are similar to natural temperate lakes, but lower than temperate agricultural impoundments. We considered the influence of lake morphometry on OC storage in our study lakes, but did not observe an inverse relationship between lake size and OC burial rate, as has been seen in some temperate lake districts. We did, however, find an inverse relation between mean water depth and OC sequestration. Despite recent cultural eutrophication and the associated shift from macrophyte to phytoplankton dominance in the Florida study lakes, overall OC burial rate increased relative to historic (pre-1950 AD) values. Lakes cover >9000 km^2^ of the Florida landscape, suggesting that OC burial in sediments amounts to as much as 1.6 Mt a^-1^. The high rate of OC burial in Florida lake sediments indicates that subtropical lakes are important for carbon sequestration and should be included in models of global carbon cycling.

## Introduction

As evidence for recent anthropogenic climate change has grown, there has been increasing interest in carbon cycling in the biosphere. Although inland waters were once viewed as short-term organic carbon (OC) storage areas or simple conduits for transport of terrestrial carbon to the coasts, their perceived role in carbon cycling has expanded, and they are now viewed as complex aquatic ecosystems that receive, transport, process and store both allochthonous and autochthonous carbon [1]. Many lakes that display negative net ecosystem production, i.e. community respiration>gross primary production, nevertheless accumulate OC in the sediments [2]. Lake sediments that accumulate organic matter are effective long-term sinks for carbon [3], and annual OC burial in lakes and reservoirs worldwide exceeds OC sequestration in ocean sediments [4]. OC burial in lakes also increases in response to human-induced eutrophication [5]. In agricultural impoundments, OC burial may be orders of magnitude greater than burial in natural inland waters [6].

Most research on OC burial in lake sediments has focused on higher-latitude temperate, boreal and Arctic water bodies, though there has been some recent work on OC burial in tropical lakes [7]. Few studies, however, have evaluated OC burial in subtropical aquatic ecosystems. Some attributes of subtropical lakes suggest that large amounts of carbon are fixed within their water columns each year. For instance, subtropical lakes in Florida are never ice-covered in winter, so they can experience high rates of primary production year-round [8]. Summer temperatures often exceed 30°C in surface waters, and temperatures rarely fall below ~13–18°C in the water column during winter. The same factors that lead to high rates of primary production, however, may lead to relatively low net carbon sequestration in sediments. For instance, high rates of decomposition may prevail at the high ambient temperatures. Furthermore, most lakes in Florida with large surface areas are shallow, z_max_ < 5 m. In such lakes, characterized by large fetch and shallow depth, the water column is wind-mixed regularly and remains oxygenated year-round. This also promotes breakdown of organic matter. But because some shallow Florida lakes are known to have thick accumulations (3–8 m) of organic sediments [9, 10], we sought to determine recent OC burial rates in some of these water bodies.

Gu et al. [11] showed an inverse relationship between latitude and δ^13^C of organic particulate material in lakes and hypothesized that carbon dynamics in lakes of warm and cold climates differ. In addition to temperature, the organic matter source influences OC degradation rate. Terrestrial (allochthonous) OC is more recalcitrant than carbon produced within the lake proper (autochthonous OC), and the former experiences slower rates of degradation [12]. Because both OC production and decomposition rates occur at high and variable rates in subtropical lakes, and may be influenced by many factors, it can be difficult to estimate long-term OC storage from results of short-term studies, e.g. daily measures of OC fixation, determined from oxygen production and respiration. Even longer-term studies, with durations of months, may lead to erroneous calculations of net, long-term OC storage. Paleolimnological techniques, however, can be applied in shallow, subtropical lakes to quantify OC storage over longer timescales. Such direct measures of OC accumulation on the lake bottom integrate time and avoid the vagaries of short-term variations in OC fixation and metabolism, which may be influenced by factors of short duration such as cloudiness, water turbulence, rainfall-mediated nutrient delivery, algal blooms, water-column oxygen depletion, etc.

Multiple paleolimnological techniques can be used to estimate OC burial in lakes. They range from the rapid, single-core method, to the intensive, whole-basin approach [13]. Recent OC burial estimates are typically derived from analysis of multiple ^210^Pb-dated sediment cores [5] and provide information about OC sequestration during the previous ~100 years, sometimes at sub-decadal temporal resolution. Alternatively, repeated bathymetric surveys can quantify recent rates of bulk and OC sedimentation by determining net sediment and OC accrual between survey dates [6]. Regardless of the technique selected, spatial heterogeneity in lake sediment deposition must be accounted for when estimating whole-lake OC storage.

**Fig 1:**
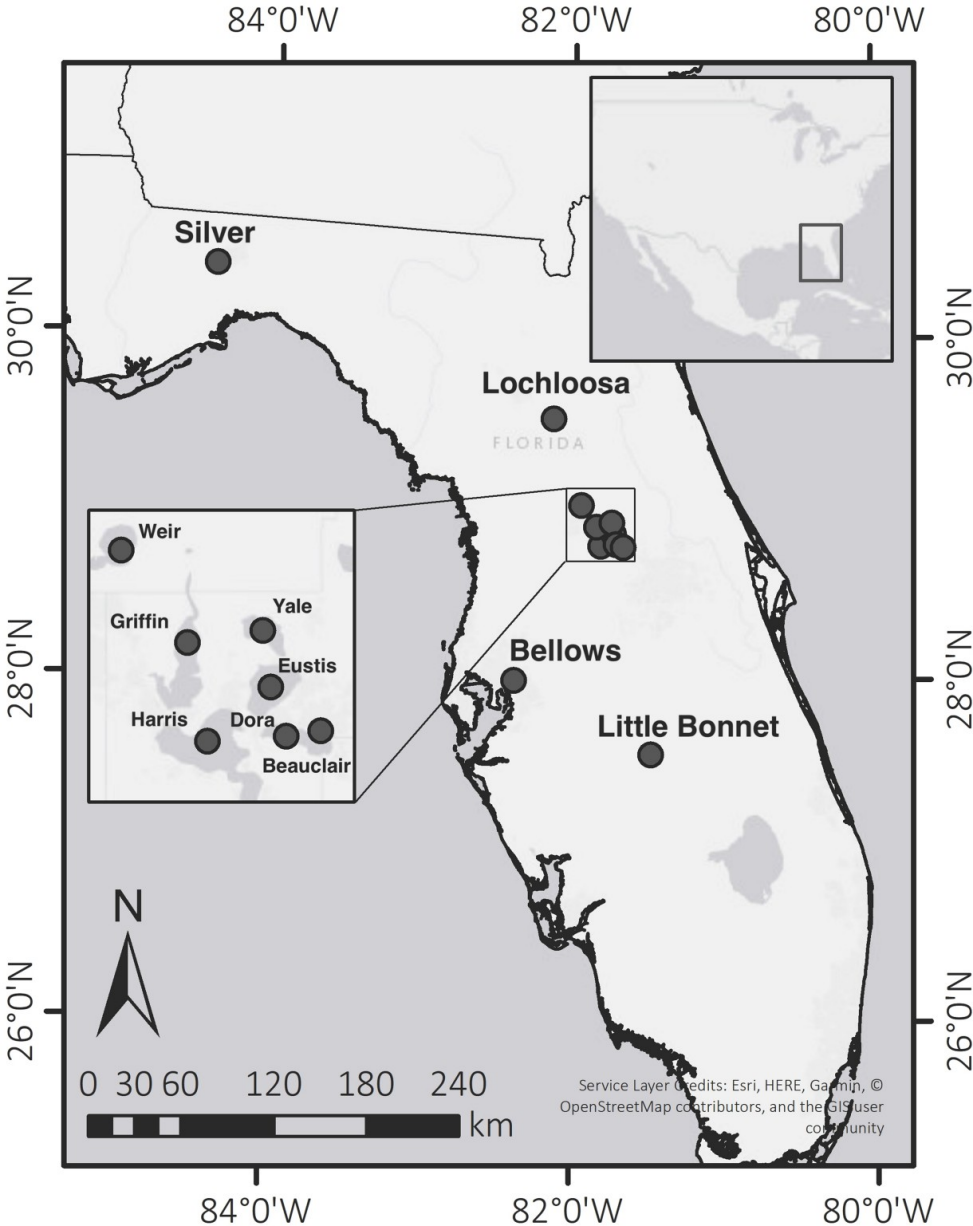
Map showing lake locations in Florida, USA. Inset maps show the entire continental USA and the Harris Chain of Lakes. Base maps include World Light Gray Base [19] and Unites States generalized [20].

We used ^210^Pb-dated sediment cores from previously published studies as well as newly collected cores to estimate rates of recent OC sequestration in 11 shallow Florida lakes (Table 1, Fig 1). We employed the whole-basin approach in Lake Lochloosa (23 km^2^) and the multiple-sediment-core approach in the other 10 lakes, which ranged in surface area from 0.1 to 75 km^2^. We used our data set of OC burial values in sub-tropical Florida water bodies to achieve three primary research objectives: 1) determine recent rates of OC burial in subtropical Florida lakes and compare the values to those from lakes at higher latitudes, 2) identify factors that influence OC burial in Florida lakes, as has been done in temperate lakes (e.g. lake size) [5, 6], and 3) assess whether there has been an increase in OC burial rates in Florida lakes as a consequence of recent cultural eutrophication.

**Table 1:** Limnological characteristics of the Florida study lakes. Data are from Florida LAKEWATCH (http://lakewatch.ifas.ufl.edu/). Surface area (SA) is in km^2^, mean water depth is in m. Total phosphorus (TP), total nitrogen (TN) and chlorophyll a (Chl *a*) are in μg/L. *Mean water depth for Little Bonnet Lake was not reported.

	DATES SAMPLED	LAT (N)	LONG (W)	SA	DEPTH	TP	TN	CHL
**BEAUCLAIR**	1990–2012	28.8	-81.7	4.4	1.7	118	3647	162
**BELLOWS**	1998–2014	28.0	-82.4	0.4	1.7	91	2270	107
**LITTLE BONNET**	1999–2015	27.6	-81.5	0.3	*	24	1934	41
**DORA**	1990–2015	28.8	-81.7	18.1	2.4	69	3280	140
**EUSTIS**	1990–2015	28.9	-81.7	31.4	2.5	34	2046	55
**GRIFFIN**	1990–2013	28.8	-81.9	38.0	2.0	59	2860	117
**HARRIS**	1990–2012	28.8	-81.8	75.8	3.3	34	1707	51
**LOCHLOOSA**	1993–2015	29.5	-82.1	22.9	1.8	67	2290	81
**SILVER**	2000–2007	30.4	-84.4	0.1	2.4	9	313	3.7
**WEIR**	1990–2015	29.0	-81.9	23.0	5.1	12	810	13
**YALE**	1990–2015	28.9	-81.8	16.4	2.8	24	1665	31

## Materials and methods

Lakes for this study were chosen to represent a range of size, nutrient concentrations, trophic state, and spatial distribution (Table 1, Fig 1). Most Florida lakes are shallow, so mean water depths were <6 m for all lakes studied. The number of cores collected in each lake was determined in earlier sediment studies that were designed to assess lake-wide deposition, and for which sediment OC data had been obtained. For each lake, soft sediment surveys were first conducted to identify optimal coring sites. For this study, Silver Lake was also cored to include a lake with a small surface area.

Sediment/water interface cores (1–2 m in length) were collected with a piston corer designed to retrieve undisturbed sediment profiles [14]. Cores were extruded in the field and sectioned at 4-cm or 5-cm intervals. These seemingly broad intervals were chosen to provide sufficient dry mass for multiple analyses, but do not compromise temporal resolution, given the relatively rapid linear sedimentation rates in these lakes. We stored the samples in low-density polyethylene cups in ice chests for transport to the laboratory. Sediment subsamples were weighed wet, dried, and re-weighed to calculate percent dry mass. Organic matter content was determined by weight loss on ignition (LOI) after combustion at 550°C for 2 h. We measured OC with a Carlo Erba NA1500 CNS elemental analyzer [15]. In cases for which OC in sediment samples was not measured directly, we converted LOI to OC by multiplying LOI by 0.469 [16]. We measured sediment total phosphorus (P) with a Bran-Luebbe auto-analyzer system, after persulfate digestion [17]. We determined sediment bulk density (g dry cm^-3^ wet) from the proportion of dry matter in wet sediment and proportions of inorganic and organic matter in dry sediment, using the equation of Binford [18].

Sediment cores were dated using ^210^Pb. We measured total and supported ^210^Pb activities, the latter estimated by activities of ^214^Pb and ^214^Bi, using low-background gamma counting with well-type intrinsic germanium detectors [21, 22]. Unsupported ^210^Pb activity was calculated as total ^210^Pb activity minus supported ^210^Pb activity. We calculated sediment ages using the constant rate of supply (CRS) model [23, 24], and propagated age errors using first-order approximations according to Binford [18].

OC burial rates at core sites were calculated by multiplying bulk sediment accumulation rates by the proportion of OC in dry sediment. We corrected OC burial estimates from individual core locations for sediment focusing [13] by multiplying the sedimentation rate by the ratio of the expected, regional unsupported ^210^Pb inventory (33.5 dpm cm^-2^) [25] to the observed unsupported ^210^Pb inventory. This correction was adjusted for cores from Lake Lochloosa, where whole-basin analysis of 13 ^210^Pb-dated cores indicated that the mean unsupported ^210^Pb inventory was lower (21.3 dpm cm^-2^) than the putative regional value.

To determine modern OC deposition rates, a beginning date of AD 1990 was chosen as the onset of “modern” deposition. This date was chosen so that OC burial would reflect changes from recent eutrophication, but not be overly influenced by dense algal abundance or lack of sediment diagenesis in surface sediments. In addition, ^210^Pb-dated sediments were divided into AD pre-1950 and post-1950 deposits to evaluate the impact of eutrophication on OC burial. AD 1950 was chosen based on the fact that many Florida lakes experienced accelerated eutrophication around that time [26–29].

## Results

**Fig 2:**
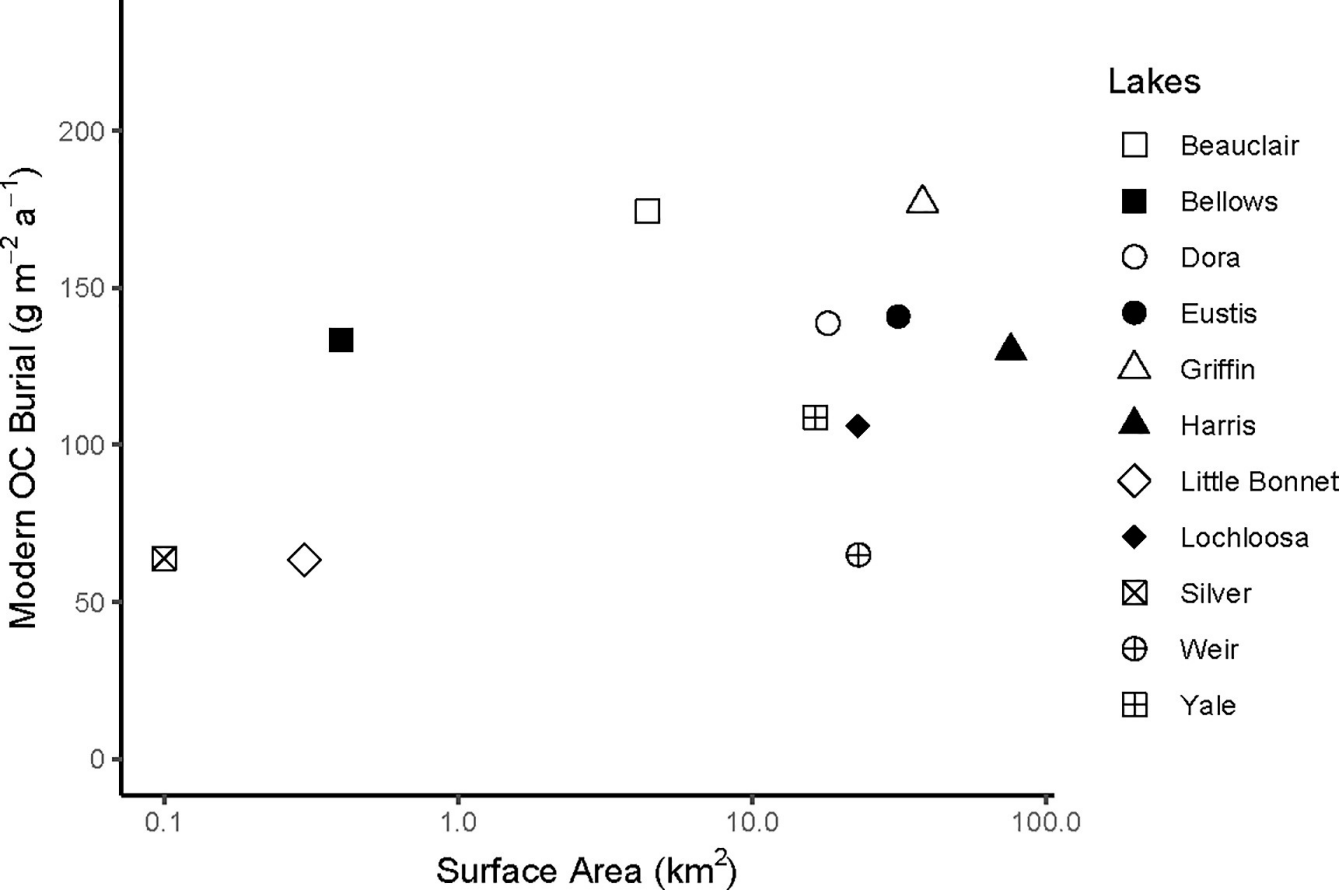
Lake surface area versus recent organic carbon (OC) burial rate for 11 shallow, subtropical Florida lakes.

**Fig 3:**
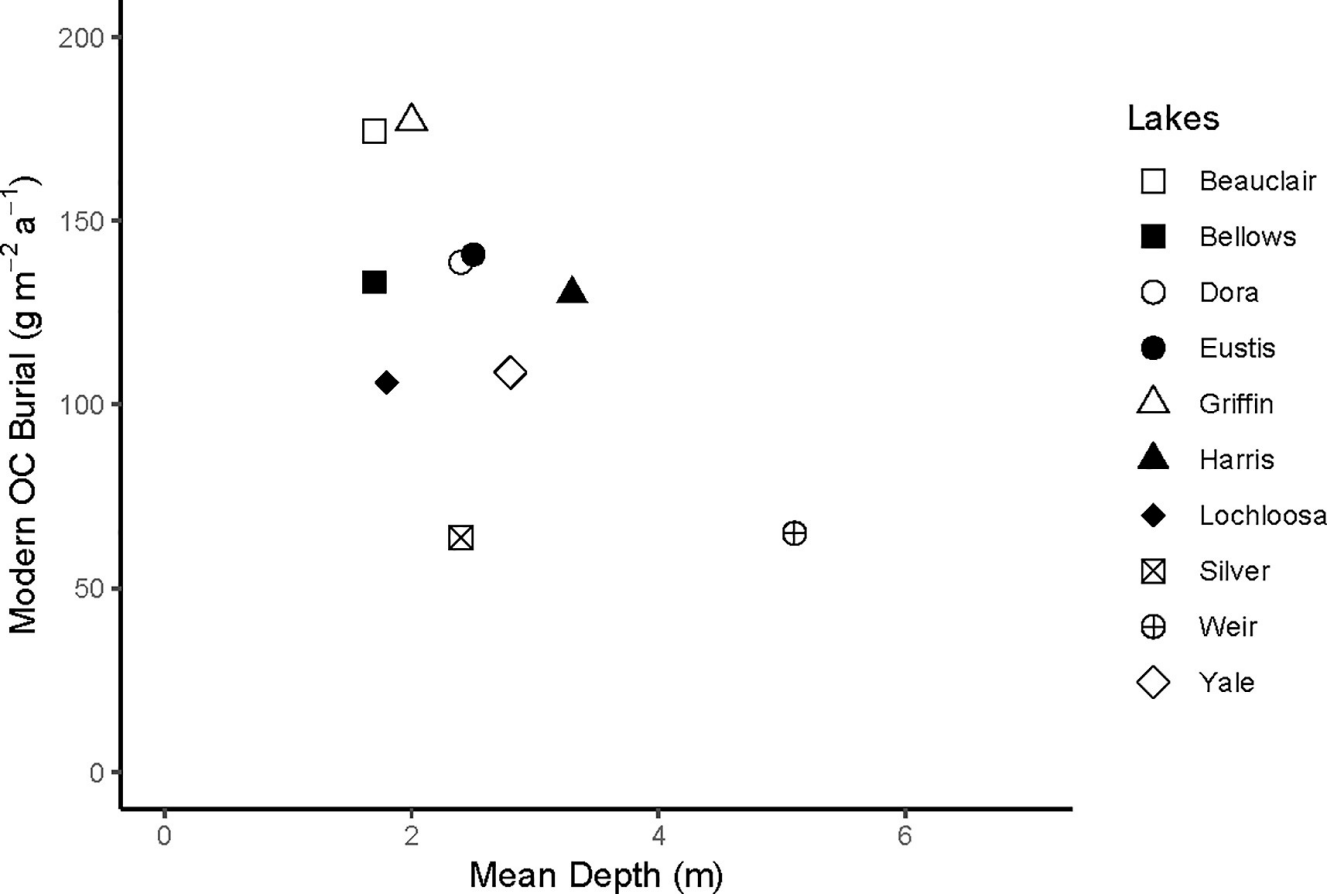
Mean depth (m) versus mean recent (post-1950) organic carbon (OC) burial rate (g OC m^-2^ a^-1^) for the 11 study lakes in subtropical Florida. Vertical bars indicate the standard deviations about the mean, based on the values derived from multiple cores in each lake.

Average recent OC burial rates (past ~150 years) in shallow Florida lakes ranged between 63 and 177 g C m^-2^ a^-1^ (Table 2). The average for all lakes studied was 118 ± 41 g OC m^-2^ a^-1^, with the minimum and maximum values from individual cores of 54 and 259 g OC m^-2^ a^-1^, respectively (Table 3). In our study lakes, OC burial rate did not decrease with increasing lake size (Fig 2), unlike the relationship reported for temperate water bodies [5, 6]. We found, instead, that the OC burial rate in Florida lakes was inversely related to lake mean depth for all but three of our lakes (Fig 3).

**Table 2:** Number of cores per lake used in this study, organic carbon (OC) content (%) and modern OC burial rates (g OC m^-2^ a^-1^) in recent sediments of the Florida study lakes.

	# of cores	g OC m^-2^ a^-1^	OC (%)	References
**Beauclair**	1	174	36.2	[27, 30]
**Bellows**	3	133	22.7	[31]
**Little Bonnet**	3	64	27.0	[32]
**Dora**	7	139	35.0	[33]
**Eustis**	7	141	31.2	[33]
**Griffin**	8	177	30.8	[34]
**Harris**	9	130	34.8	[27, 30]
**Lochloosa**	13	106	28.2	[35]
**Silver**	1	64	26.7	This study
**Weir**	3	65	32.2	[27, 30]
**Yale**	3	109	34.3	[27, 30]

**Table 3:** Organic carbon (OC) data for individual cores used in this study and means for each lake.

Lake	Core	Date of Collection	Modern Burial Oldest Date	FF-Corrected^@^ Modern OC Burial g m^-2^ a^-1^	After 1950 Oldest Date	FF-Corrected After 1950 OC Burial g m^-2^ a^-1^	Before 1950 Youngest Date	Before 1950 Oldest Date	FF-Corrected Before 1950 OC Burial g m^-2^ a^-1^
**Beauclair**	2*^+^	**1999**	**1989**	**174.3**	**1956**	**129.6**	**1956**	**1895**	**54.8**
**Bellows**	B1	2010	1999	148.7	1948	209.2	1948	1878	100
	B4	2010	1995	108.2	1958	161.2	1941	1892	57.4
	B5	2010	2001	143.0	1956	172.4	1942	1912	101.8
	**Mean**			**133.3**		**180.9**			**86.4**
**Little**	B1	2008	1991	68.2	1949	53.4	1949	1886	246
**Bonnet**	B2	2008	1990	63.5	1949	109.2	1949	1921	64.5
	B3	2008	1993	58.4	1947	39.5	1947	1934	44.4
	**Mean**			**63.4**		**67.4**			**118.3**
**Dora**	3H	1995	1984	141.4	1951	109.4	1951	1886	46.9
	6H	1998	1988	134.4	1953	118.4	1953	1896	97.5
	10H	1995	1985	128.6	1951	93.8	1951	1887	36.2
	12H	1998	1988	140.2	1958	96.2	1945	1888	26.6
	14H	1995	1985	131.4	1951	106	1951	1927	56.3
	21H	1996	1987	142.4	1949	105.7	1949	1891	54.2
	22H	1996	1985	152.3	1949	129.5	1949	1917	58.1
	**Mean**			**138.7**		**108.4**			**53.7**
**Eustis**	3H	1998	1987	137.4	1951	154.6	1951	1890	92.8
	11H	1995	1986	152.1	1955	91.8	1949	1887	44.8
	13H	1995	1986	158.6	1951	131.8	1951	1885	55
	16H	1995	1985	122.5	1953	93.8	1953	1895	70.9
	27H	1998	1988	122.4	1958	76.2	1931	1882	22.2
	28H	1996	1985	150.5	1952	116.7	1952	1888	51.7
	29H	1996	1985	142.1	1953	105.6	1953	1889	46.9
	**Mean**			**140.8**		**110.1**			**54.9**
**Griffin**	2H	1994	1984	143.2	1952	109.7	1952	1886	42.3
	3H	1995	1985	70.2	1952	127.2	1952	1889	56.6
	7H	1995	1985	259.3	1950	182.0	1950	1886	46.5
	16H	1995	1982	176.6	1953	217.2	1953	1889	84.4
	26H	1994	1985	148.1	1955	110.9	1955	1887	60.3
	43H	1995	1986	200.2	1954	143.1	1954	1891	74.2
	44H	1995	1985	241.5	1951	159.3	1951	1903	40.8
	**Mean**			**177.0**		**149.9**			**57.9**
**Harris**	1	1999	1992	120.8	1974	101.8	1938	1899	180.5
	2	1999	1991	136.4					
	3	1999	1990	120.1	1947	74.4	1947	1920	39.1
	4	1999	1992	126.4	1958	88.7	1945	1895	72.6
	5	1999	1987	121.3	1950	133.5	1950	1886	141.2
	6	1999	1991	112.1	1950	72.6	1950	1898	53.4
	7	1999	1990	131.0	1952	76.7	1952	1909	20.2
	8	1999	1989	143.8	1950	105.8	1950	1888	106.8
	9	1999	1991	158.5	1951	112	1951	1897	57.8
	**Mean**			**130.0**		**95.7**			**84.0**
**Lochloosa**	13 cores	**2006**	**1997**	**106.0**					
**Silver**	**1**	**2011**	**1993**	**63.8**	**1967**	**56.8**	**1931**	**1881**	**20.9**
**Weir**	1R	1999	1990	60.2	1953	46.2	1953	1893	53.9
	2R	1999	1986	53.8	1961	43.8	1946	1887	65.1
	3R	1999	1986	80.9	1960	70.6	1947	1889	41.6
	**Mean**			**65.0**		**53.5**			**53.5**
**Yale**	1	1999	1990	102.7	1953	64.3	1953	1899	28.1
	2	1999	1992	124.4	1968	89.6	1940	1889	21.3
	3	1999	1987	99.1	1955	70.8	1938	1888	75.2
	**Mean**			**108.7**		**74.9**			**41.5**

*Means used for figures in bold.

^+^For Lake Beauclair and Silver Lake only one core was used. As a result, the bold number for these two lakes reflects the calculated OC measurements for the single core instead of a mean.

^@^ FF-Corrected are OC storage values corrected for sediment focusing.

**Fig 4:**
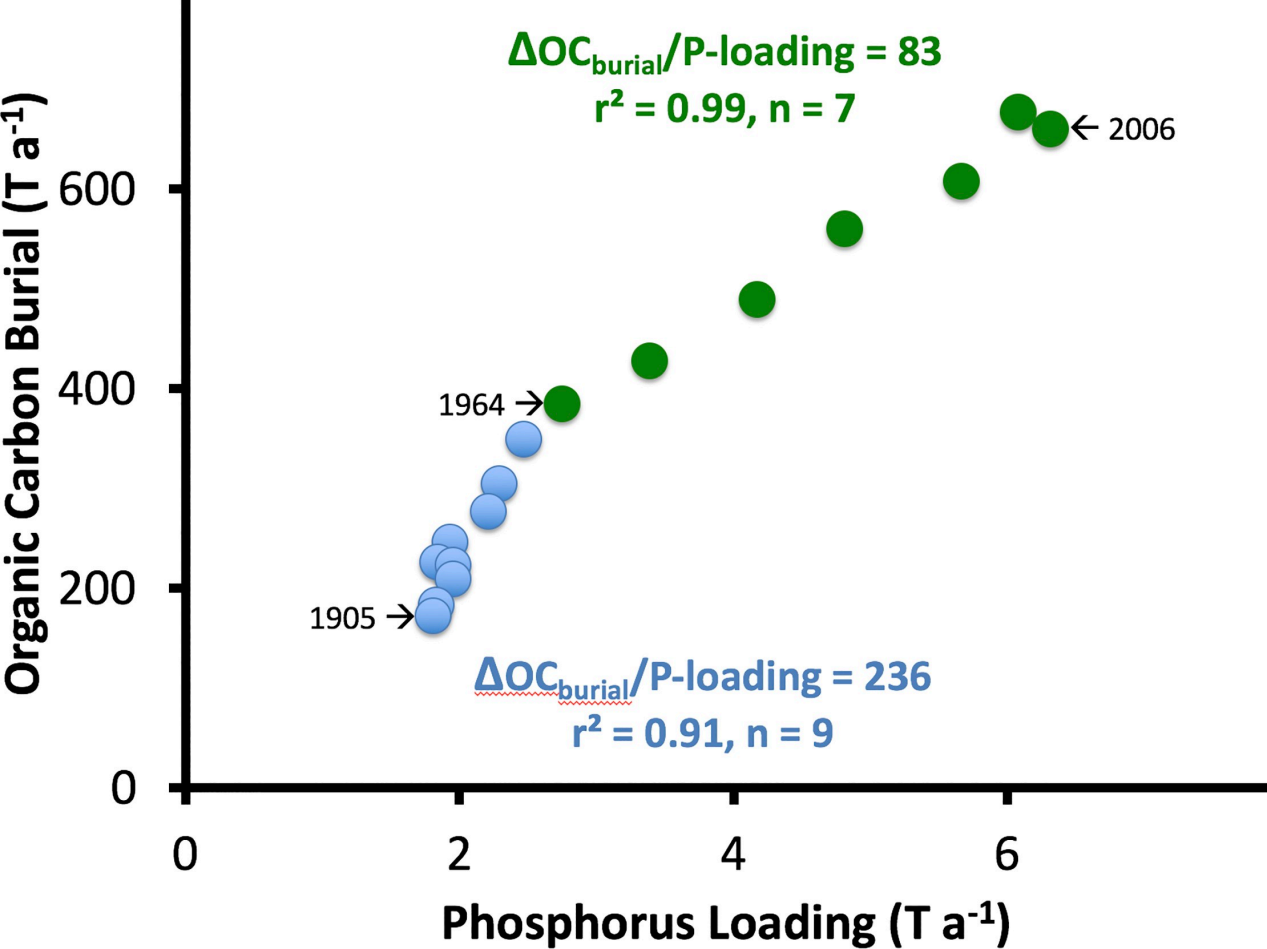
Historical phosphorus loading versus organic carbon (OC) burial in Lake Lochloosa, Florida. Values were determined with the whole-basin, mass-balance approach, using 13 ^210^Pb-dated sediment cores [35]. In that study, whole-basin OC burial was estimated by extrapolating measured values only over the area of the lake that contained recent sediments (~7 km^2^, or ~31%). Before 1964 the lake was macrophyte-dominated (blue circles) and after 1964 the lake was phytoplankton-dominated (green circles). Values corresponding to the basal date (1906), the date of the switch in primary producer community structure (1964), and the core collection date (2006) are indicated with arrows. OC burial was lower, but more efficient relative to P loading (i.e. ΔOC_burial_/P_loading_) during earlier times when the lake was macrophyte-dominated, compared to more recent times when the lake was phytoplankton-dominated.

Kenney et al. [35] used the whole-basin, mass-balance approach to determine historical P loading in Lake Lochloosa. Comparison of historical P loading to historical OC burial in Lake Lochloosa showed that OC burial increased through time as a two-phase linear function of P loading (Fig 4). When P loading was lower in the past (<2.7 T a^-1^), the lake was macrophyte-dominated and ΔOC_burial_/P_loading_ was 236. As P loading increased more recently (>2.7 T a^-1^), the lake became phytoplankton-dominated and ΔOC_burial_/P_loading_ declined to 83.

**Fig 5:**
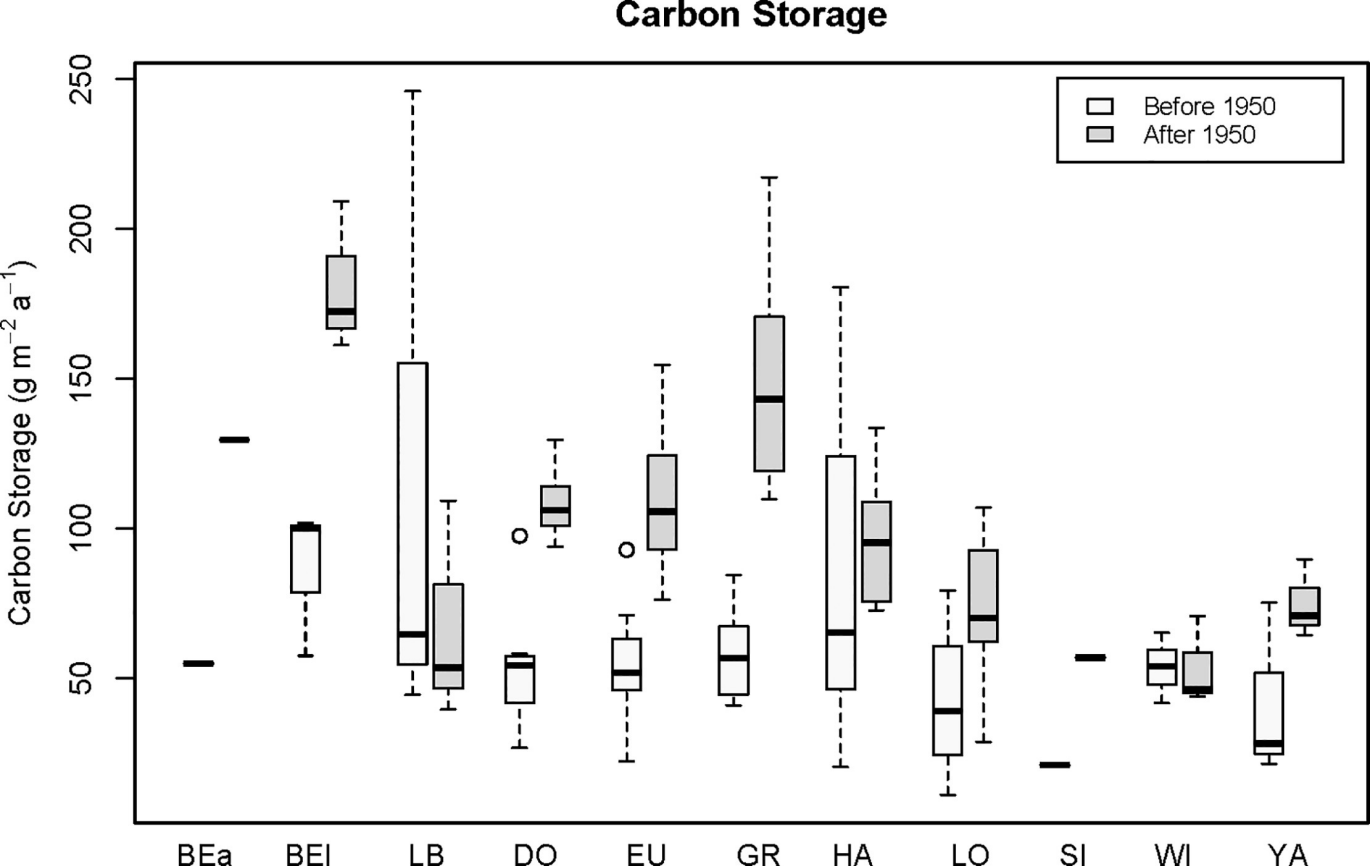
Boxplots of pre-1950 and post-1950 mean organic carbon (OC) storage rates (g OC m^-2^ a^-1^) in sediments of 11 study lakes in subtropical Florida. Dates were determined by ^210^Pb measurements and application of the CRS model. Dates before 1950 include all datable core sections pre-1950. Lake abbreviations are as follows: Beauclair (BEa), Bellows (BEI), Little Bonnet (LB), Dora (DO), Eustis (EU), Griffin (GR), Harris (HA), Lochloosa (LO), Silver (SI), Weir (WI), and Yale (YA). Values for Lakes Beauclair and Silver reflect storage rates derived from a single core in each lake. The open circles above the Lake Dora and Eustis boxplots are points that fell outside the range of the upper quartile plus 1.5 times the interquartile distance.

With the exception of Lakes Little Bonnet and Weir, nine of the 11 study lakes showed increased OC burial during recent decades (AD 1950 to present) (Fig 5). Among all the lakes, OC burial after 1950 averaged 92 ± 36 g C m^-2^ a^-1^, whereas the pre-1950 average was 61 ± 25 g C m^-2^ a^-1^, indicating a 51% increase across the time period. The timing of this increase in OC burial corresponds to the period of accelerated eutrophication in each system, and in many other shallow lakes throughout Florida [26–29].

## Discussion

Sediments of shallow, subtropical Florida lakes are effective OC sinks and their OC burial rates (63–177 g C m^-2^ a^-1^) rank among the largest measured in natural aquatic ecosystems in the world [5, 6, 36]. Because lakes cover >9000 km^2^ of Florida’s area [37], when we apply our estimates of OC sequestration to water bodies throughout the state, we calculate a state-wide rate of total OC burial in lakes of 0.6–1.6 Mt a^-1^. Spatially heterogenous sediment distribution within some shallow Florida lakes [38] probably limits the area over which OC burial occurs. Nevertheless, Florida has >11,000 km^2^ of inland aquatic ecosystems (www.StateofFlorida.com), and if we include lakes, wetlands, rivers and springs, combined OC burial across all these freshwater ecosystems in Florida certainly amounts to nearly 2 Mt a^-1^. Even if our first-order approximation of OC sequestration in Florida water bodies is subject to error, some of which may be related to extrapolation to such a large area [7], the result indicates the importance of shallow subtropical aquatic ecosystems in global OC sequestration.

The mean OC burial rate for all 11 of the Florida study lakes was 118 g C m^-2^ a^-1^. This rate is lower than tropical systems of the Amazon Basin (266 g C m^-2^ a^-1^) [7] and temperate agricultural impoundments (>1000 g C m^-2^ a^-1^) [6]. Nevertheless, the numbers from Florida’s subtropical lakes are higher than values measured in many Arctic (6 g C m^-2^ a^-1^) [39], boreal (2 g C m^-2^ a^-1^) [40], temperate (33 g C m^-2^ a^-1^) [41], and tropical (24 g C m^-2^ a^-1^) [42] systems. Our subtropical lakes yielded OC burial values similar to those from temperate lakes in agricultural landscapes (64–200 g C m^-2^ a^-1^) [5].

Many factors influence OC burial in lakes. Such factors can be unique to individual water bodies and/or characteristic of entire lake regions. These can include OC fixation rates, which are dependent on nutrient concentrations, as well as conditions that affect bacteria-mediated decomposition of organic matter (e.g. temperature, oxygen, organic matter concentration). The specific composition of the OC and how the OC interacts with the mineral matrix [43] can also affect OC storage in the sediment.

The mean OC content of shallow Florida lake sediments is large (~30%) relative to OC concentrations measured in many inland aquatic ecosystems elsewhere. For instance, the OC content in sediments of subtropical Yangtze River floodplain lakes is <2% [44]. In Amazonian floodplain lakes, OC represents <10% of the sediment mass [7], and in nearshore marine environments and fjords, typical values are ~2.5% [45]. Many freshwater impoundments have OC concentrations in sediments of ~5% [6], and Sobek et al. [39] reported a value of <6% for natural lakes in other regions. Sobek et al., however, reported relatively high OC content (~25%) in sediments of three oligotrophic Swedish boreal lakes that accumulate predominantly allochthonous organic matter [39].

Subtropical Florida lakes, particularly those with large surface area, are shallow. Most have z_max_ <5 m and the organic matter that accumulates in them is predominantly of autochthonous origin [46–50]. The difference between sources of OC in sediments of boreal Swedish lakes (terrestrial) and subtropical Florida lakes (aquatic) is not surprising, given the differences in climate, soil, topography and vegetation between the two regions. What is surprising, however, is the fact that OC burial rates in Florida lakes are larger than those in boreal lakes, where allochthonous OC in the sediment dominates. One might expect larger values in the boreal lakes, given the more recalcitrant nature of the allochthonous organic matter [12]. In addition, whereas some temperate lakes report similar OC burial rates [5], the mechanism of OC accumulation appears to differ, with terrestrial carbon constituting a larger portion of OC burial increases in temperate regions.

The OC content of Florida lake sediments is an order of magnitude larger than that of most lake sediments elsewhere, but the OC burial rate is within the range of OC burial rates for many lakes. Lower delivery of terrigenous inorganic matter to Florida lakes probably explains the discrepancy between OC concentrations in Florida lakes and lakes in other regions. The landscape of peninsular Florida displays low topographic relief and upland soils are typically sandy and well-drained [51], so common mechanisms for delivery of terrigenous material to lakes elsewhere, i.e. colluviation and alluviation, are largely absent in Florida. The relatively small contribution of allochthonous inorganic material to Florida lake sediments is exemplified by the Holocene sediment record from Lake Harris, in which the combined masses of organic matter, biogenic silica and biogenic carbonate account for 75% of the total sediment mass [49]. The organic-rich sediment dry mass is about 30% OC, but <25% is allochthonous inorganic matter. It would require a ~40-fold increase in the burial rate of allochthonous inorganic matter in Lake Harris to reduce the OC content of the sediment to a value similar to that in many lakes elsewhere (i.e. ~3%). Likewise, paleolimnological records from many Florida lakes show C/N molar ratios <12 during periods when the lakes were eutrophic or hypereutrophic, indicating algae and cyanobacteria were the primary constituents of sedimented organic matter [26, 52].

In north-temperate lakes, the OC burial rate decreases with increasing lake size [6]. This observation was attributed to greater inputs of allochthonous OC, relative to autochthonous OC, in smaller lakes. This trend, however, was not encountered in our study lakes (Fig 2). Because the flat landscape and well-drained soils of Florida [51] are not conducive to delivery of terrestrial material to lakes, input of allochthonous OC to Florida lakes is negligible, and there is thus no relationship between lake size and OC burial. Instead, OC burial rates in Florida lakes are influenced by autochthonous production, which is dictated by nutrient concentrations, and lake mean depth (Fig 3), with lower rates of OC sequestration at larger depths.

Recent increases in OC sequestration in temperate lake systems appear to result from a combination of greater terrigenous inputs and eutrophication [5], but cultural eutrophication appears to be the main factor driving increased OC burial in Florida lakes. The larger and shallower lakes in our study have the highest OC burial rates (Figs 2 and 3). The large surface area and shallow depth of many Florida lakes enables frequent sediment resuspension during wind events. During these events, sediment phosphorus (P) is returned to the water column (internal loading) in forms required by primary producers [53]. Because of frequent internal release of legacy sediment P, efforts to reduce external P inputs may have little impact on eutrophication [54]. For example, Florida’s Lake Apopka maintained eutrophic conditions, despite more than a decade of aggressive management strategies designed to reduce external P loading [55].

In Lake Lochloosa, the whole-basin, mass-balance approach shows that the OC burial rate increased with greater P loading, but as the lake shifted from macrophyte to phytoplankton dominance, the ratio of OC burial relative to P loading (i.e. ΔOC_burial_/P_loading_) decreased. Macrophytes have more structural carbon than do phytoplankton, so the observed ratios of OC burial relative to P loading were consistent with the expected elemental stoichiometry of the prevailing primary producer community structure at the time the sediment was deposited [56]. The positive relationship between OC burial and P loading reflects the control of P input on primary productivity in lakes [57]. The observed link between eutrophication (i.e. greater P loading) and increased OC burial in subtropical Lake Lochloosa suggests that as lakes in cooler climates warm [58] and experience eutrophication [59], OC burial in those systems will also increase.

Measured, long-term OC burial rates in Florida lakes, determined by paleolimnological analysis, suggest that net OC production over short timescales is so small that measurements of net primary productivity in the water column (e.g. light-dark bottle or day/night measures of oxygen production) are inadequate to determine if these lakes are autotrophic (production>respiration) or heterotrophic (respiration>production) [60]. For instance, using the sediment records from our study lakes, we found the greatest OC burial rate in shallow (z_mean_ = 2 m) Lake Griffin (~177 g OC m^-2^ a^-1^). That value, converted to an hourly, volumetric rate in a well-mixed, 2-m water column, is equal to about 0.02 mg OC L^-1^ hr^-1^. This estimate of net volumetric OC production rate represents the largest value in our data set, and most Florida lakes probably have even lower rates of short-term OC production. Because net OC production is near zero, and there is natural variability in the system, on any given day such a lake may appear to be either autotrophic or heterotrophic, depending on the short-term weather conditions. Dated sediment cores integrate long time spans and avoid the pitfalls of attempting to estimate OC sequestration in lakes using short-term analyses of water column variables that are subject to considerable natural variability.

In nine of 11 lakes for which we compared temporal shifts in OC accumulation, all but two showed an increase in OC burial rate after ca. AD 1950 (Fig 4), Lake Weir and Lake Little Bonnet being the only exceptions. Like Lake Lochloosa, the other study lakes experienced increases in primary productivity, which caused increased OC burial [27, 30, 33, 34]. We assume that the increase in labile OC burial resulted in an increase in bacterial degradation during the same period, suggesting that the original OC deposition was greater than the net OC burial measured, i.e. some OC was lost to diagenesis. Nevertheless, increases in OC sedimentation outpaced the degradation process, suggesting that productivity is the process that enables shallow subtropical lakes to permanently sequester OC in the sediments. The average OC burial rate calculated for the pre-1950 period in our study lakes (61 g OC m^-2^ a^-1^, Table 3) is still greater than the Arctic, boreal, temperate, subtropical, and tropical systems mentioned above [39–42].

## Conclusions

We used ^210^Pb-dated sediment cores from 11 shallow Florida lakes to estimate recent OC burial in these subtropical aquatic ecosystems. Sediments in Florida lakes have high OC content (~30%) relative to most natural inland water bodies, and are effective sinks for OC, with recent net burial rates of 63–177 g C m^-2^ a^-1^. Considering that Florida lakes cover an area >9000 km^2^, overall OC burial in these systems is large (~1.6 Mt a^-1^). Shallow Florida lakes differ from north-temperate lakes in that Florida’s subtropical water bodies did not display an inverse relationship between lake size and OC burial rate, as was encountered farther north. Shallower Florida lakes stored OC at higher rates than did deeper Florida lakes. OC burial rates in Florida lakes increased since ca. 1950 as a consequence of eutrophication, and the shift from macrophyte to phytoplankton dominance reduced the ratio of OC burial relative to phosphorus loading.

## Supporting information

S1 Table Organic carbon storage for Silver Lake, Florida. (PDF)
