# Supplementary material for: Organic carbon sequestration in sediments of subtropical Florida lakes
Source: PLoS One. 2019 Dec 13;14(12):e0226273. doi: 10.1371/journal.pone.0226273 (PMC6910687; doi:10.1371/journal.pone.0226273)
Supplement: S1 Table — (PDF) [file pone.0226273.s001.pdf]

Core Location: Apalachicola Forest, Fl
Core ID: SL-2011
Coring Date: 8-Jan-11

Max Depth (cm)	Fraction Dry	Fraction Organic	Rho g/cm3	Cummulative Mass g/cm2	Pb-210 dpm/g
4	0.059	0.566	0.061	0.245	37.5
8	0.067	0.552	0.069	0.521	27.4
12	0.066	0.542	0.068	0.792	18.2
16	0.048	0.625	0.049	0.988	10.3
20	0.047	0.633	0.049	1.182	3.9
24	0.049	0.661	0.050	1.384	2.6
28	0.049	0.657	0.050	1.585	2.0
32	0.052	0.657	0.054	1.799	**n.d.**
36	0.053	0.672	0.055	2.018	**n.d.**
40	0.055	0.663	0.056	2.243	2.0
44	0.051	0.676	0.052	2.452	
48	0.049	0.685	0.050	2.652	
52	0.052	0.677	0.054	2.867	
56	0.054	0.687	0.055	3.087	
60	0.050	0.679	0.052	3.294	
64	0.052	0.683	0.053	3.506	
68	0.050	0.688	0.052	3.713	
72	0.051	0.694	0.053	3.923	
76	0.052	0.676	0.054	4.138	
80	0.052	0.688	0.054	4.353	
84	0.052	0.699	0.054	4.567	
88	0.053	0.694	0.054	4.783	
92	0.055	0.691	0.056	5.009	
96	0.054	0.665	0.055	5.230	
100	0.053	0.661	0.055	5.449	
104	0.056	0.674	0.057	5.677	
108	0.062	0.710	0.063	5.930	
112	0.060	0.708	0.062	6.178	
116	0.058	0.701	0.060	6.417	
120	0.059	0.699	0.061	6.659	

**A(o)=**	18.5	dpm/cm2
**±1s error =**	0.672	
**variance =**	0.460	

**Pb-210 1s error**	**Ra-226 dpm/g**	**Ra-226 1s error**	**Cs-137 dpm/g**	**Cs-137 1s error**	**Excess Pb-210 dpm/g**	**Excess Pb-210 1s error**	**Date at Max Depth**
1.2	4.6	0.6	8.174	0.334	33.1	1.4	1992.5
1.0	7.0	0.5	8.933	0.321	20.5	1.1	1967.4
0.9	6.3	1.1	6.835	0.309	12.0	1.4	1930.9
0.5	4.2	0.5	1.608	0.114	6.2	0.7	1881.1
0.4	2.3	1.2	1.134	0.110	1.7	1.2	
0.3	3.5	0.4	1.541	0.127	**0.0**	0.6	
0.4	3.4	0.3	0.980	0.120	**0.0**	0.5	
**n.d.**	**n.d.**	**n.d.**	**n.d.**	**n.d.**	**n.d.**	**n.d.**	
**n.d.**	**n.d.**	**n.d.**	**n.d.**	**n.d.**	**n.d.**	**n.d.**	
0.3	1.8	0.8	0.426	0.068	0.2	0.9	

<-- Total inventory of excess Pb-210 (compare to 30)

Date 1s error	Mass Sedimentation Rate mg/cm2/yr	Mass Sedimentation Rate 1s error
2.1	13.3	0.8
3.5	11.0	1.0
6.7	7.4	1.4
27.8	3.9	1.6
